# The association between social engagement and depressive symptoms in middle-aged and elderly Chinese: A longitudinal subgroup identification analysis under causal inference frame

**DOI:** 10.3389/fnagi.2022.934801

**Published:** 2022-09-01

**Authors:** Yuhui Yang, Yemian Li, Peng Zhao, Jingxian Wang, Baibing Mi, Leilei Pei, Yaling Zhao, Fangyao Chen

**Affiliations:** ^1^Department of Epidemiology and Biostatistics, School of Public Health, Xi'an Jiaotong University Health Science Center, Xi'an, China; ^2^Department of Radiology, First Affiliated Hospital of Xi'an Jiaotong University, Xi'an, China

**Keywords:** social engagement, depressive symptoms, middle-aged and elderly Chinese, subgroup identification, causal inference

## Abstract

**Background:**

Studies have suggested that there is a significant association between social engagement and depression symptoms. However, this association may differ in people with different features such as different sociodemographic characteristics and health conditions.

**Methods:**

Research data were obtained from the CHARLS database. The causal inference was performed with the propensity score. We used the linear mixed-effects model tree algorithm under the causal inference frame for subgroup identification analysis.

**Results:**

We included 13,521 participants, and the median follow-up time is 4 years. Under the casual inference frame, the association between social engagement and depression symptoms is confirmed for all included individuals (OR = 0.957, *P* = 0.016; 95%CI: 0.923–0.992). Using the linear mixed-effects model tree, we found two subgroups, including middle-aged and elderly residents who live in rural areas with <6 h of sleep and those living in urban areas, could benefit more from social engagement. After using the propensity score method, all the two subgroups selected are statistically significant (*P* = 0.007; *P* = 0.013) and have a larger effect size (OR = 0.897, 95%CI: 0.830–0.971; OR = 0.916, 95%CI: 0.854–0.981) than the whole participants. As for sex difference, this associations are statistically significant in male (OR: 0.935, *P* = 0.011, 95%CI: 0.888–0.985) but not in female (OR: 0.979, *P* = 0.399, 95%CI: 0.931–1.029).

**Conclusions:**

Our findings indicate that social engagement may reduce the risks of depressive symptoms among all individuals. The identified subgroups of middle-aged and elderly residents who live in rural areas with <6 h of sleep and those who live in urban areas may benefit more from the social engagement than the whole participants.

## Introduction

The latest statistics of the World Health Organization (WHO) showed that about 322 million people suffered from depression worldwide, and the peak incidence appeared among the elderly (World Health Organization., 2017). Nearly half of the people with depressive symptoms lived in Southeast Asia and the Western Pacific regions (World Health Organization., 2017). China is the most populous country in the Asia Pacific region; with the rapid aggravation of China's aging society, the number of senior citizens with depressive symptoms (DSs) may further increase in the future (Ren et al., 2020). DS not only contributes to a bad mood, negative attitudes, poor sleep, and quality of life (Cui, 2015) but also increases suicide rates (Dong et al., 2018). It has been confirmed that DS can cause lower working efficiency and higher medical costs, resulting in huge socioeconomic losses (Murray and Lopez, 1996). Therefore, it is urgent to find effective methods to prevent and treat DS.

Current treatments for patients with confirmed DS mainly include antidepressants and psychotherapy (Schuch and Stubbs, 2019). Although antidepressants are effective for about half of the patients (Pigott, 2015), all available antidepressants have side effects of various degrees, such as weight gain, increased diabetes risk, and sexual dysfunction (Schuch and Stubbs, 2019). Meanwhile, psychological therapies, such as cognitive behavioral therapy, have limited effects on the treatment of DS (Cuijpers et al., 2014). Therefore, the way to treat depressed patients after the fact is far less helpful than preventing their disease from the beginning.

Many studies have indicated that social engagement (SE) was associated with a low risk of DS (Glass et al., 2006; Isaac et al., 2009; Lou et al., 2013). As early as 2006, Glass et al. used longitudinal data from a cohort study of the New Haven elderly population to investigate whether SE could prevent the elderly from developing depressive symptoms (Glass et al., 2006). Their results indicated that SE was negatively associated with DS (Glass et al., 2006). In long-term residential care settings, Lou et al. analyzed six waves of data collected in the Hong Kong Longitudinal Study and found that a higher level of SE was associated with fewer DS (Lou et al., 2013).

However, the associations between SE and DS mentioned above were based on the estimation of average effects at a general population-wide level. However, in fact, the association between SE and DS tends to differ across subgroups with different characteristics (different sociodemographic characteristics, health conditions, etc.). Furthermore, several longitudinal studies have already suggested that the association between SE and DS was limited to specific populations (Takagi et al., 2013; Hajek et al., 2017). For example, evidence from a multicenter prospective cohort study in Germany showed that SE was associated with decreased DS only in women, but not men (Hajek et al., 2017).

Since SE is associated with a low risk of DS, promotion of SE can provide a protective effect of preventing and mitigating the initiation and progression of DS at the lowest cost (Solomonov et al., 2019; Bae, 2021). However, as the studies mentioned above, the effect of SE is not homogenous for all subgroups of people. Therefore, for the conduction of effective and precise prevention of the development and progression of DS, it is necessary to detect those subgroups of people with a stronger association between SE and low risk of DS. However, few studies paid much attention to this issue. In this case, the methodology of subgroup identification might be a useful approach to achieve this purpose since it can identify those specific subgroups with a stronger association between SE and lower DS risk.

In addition, for observational studies, the control of confounders is very important, and the results obtained without control of confounders are likely to be misleading (Streeter et al., 2017). The presence of confounders would influence the estimated association between SE and DS and lead to misleading results. Therefore, in order to properly estimate the association between SE and DS in different subgroups, the control of confounders must be taken into account, and one of the most effective ways to control confounders at present is the frame of causal inference.

Therefore, in this study, we aim to (1) investigate whether SE is associated with DS in the middle-aged and elderly Chinese population and (2) identify whether there are subgroups of middle-aged and elderly residents in China who show stronger or weaker patterns of association between SE and DS, while possible confounding variables are controlled by using a causal inference framework.

To take the time-constant heterogeneity into account, we use longitudinal data from the China Health and Retirement Longitudinal Survey (CHARLS) database (Zhao et al., 2014) instead of cross-sectional data to obtain more reliable results (Hajek et al., 2017). Since the data are longitudinal, we have chosen the generalized linear mixed-effects model tree (GLMM tree) algorithm to perform subgroup identification. In this study, SE and DS were defined as continuous variables, and all analysis was conducted under the framework of causal inference to ensure the correctness of the conclusion.

## Methods

### Data and sample

The data were obtained from the China Health and Retirement Longitudinal Survey (CHARLS) database (https://charls.charlsdata.com/pages/data/111/zh-cn.html). The CHARLS study was approved by the Biomedical Ethics Review Committee of Peking University in June 2008. All participants signed an informed consent form when participating. Zhao et al. (2014) have provided more detailed information about the design and implementation of the CHARLS study (Zhao et al., 2014).

Our study used baseline (2011) (Zhao et al., 2013) and three-year follow-up data (2013, 2015, and 2018) (Zhao et al., 2014). More specifically, in order to take the “longitudinal effect” of SE on DS into account, the DS outcome from the waves of 2013, 2015, and 2018 was used. And that the SE from waves baseline, 2013 and 2015 were used.

Missing data are inevitable for cohort studies, and it is not recommended to be imputed when it appears in the primary outcome and key research factors (Engels and Diehr, 2003). Therefore, we selected cases that had complete data in SE and DS and imputed the missing data in other variables.

**Figure 1:**
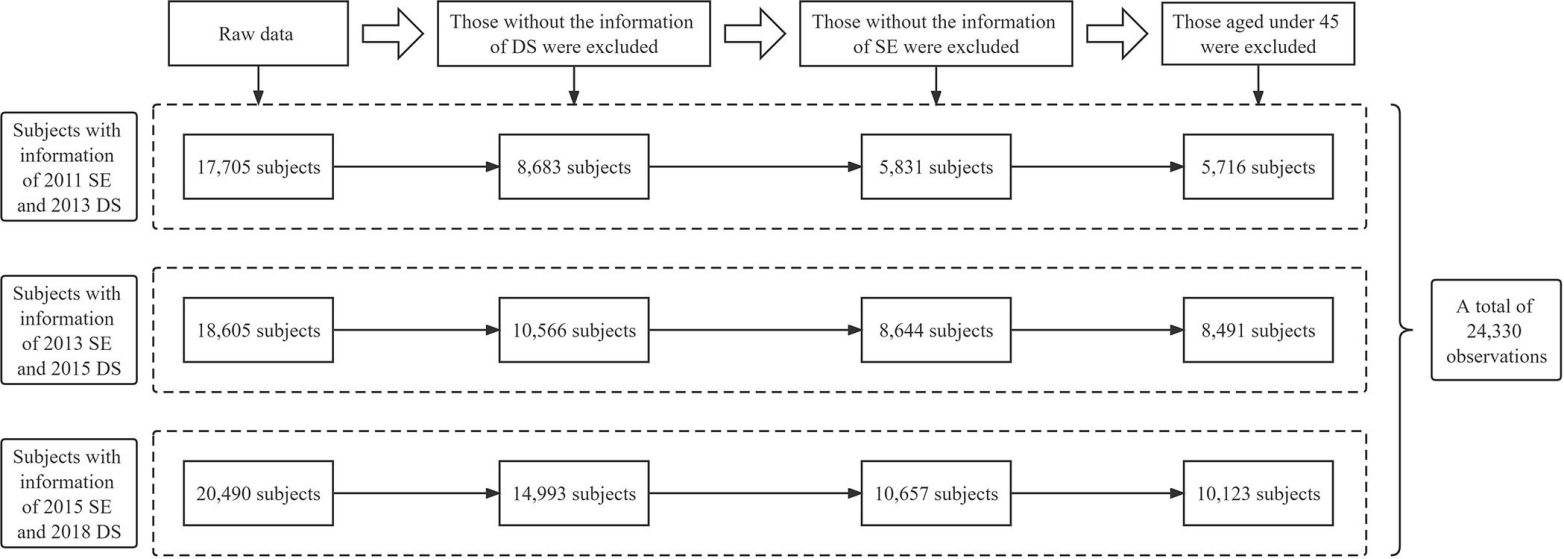
Flowchart of the data management process.

Thus, based on the requirement of the model and our research targets, we included participants according to the following criteria: (1) providing complete information about SE and DS during follow-ups; (2) aged over 45. For more detailed information, see Figure 1.

### Measurement

#### Measurement of depressive symptoms

The Center for Epidemiological Studies Depression Scale-10 (CES-D-10), effective measurement of depressive symptoms in Chinese people (Cheng et al., 2016), was used in the CHARLS study. The scale consisted of 10 items and measured the feelings and behaviors of participants. Respondents were required to answer the frequency of symptoms described in each item in the past week. Each item has four options: (1) rarely or none of the time (<1 day); (2) some or a little of the time (1-2 days); (3) occasionally or a moderate amount of the time (3–4 days); (4) most or all of the time (5–7 days), corresponding to 0–3 points, among which “I felt hopeful about the future” and “I was happy” are reverse scoring problems. The total score range of the scale is 0–30. In our study, we regarded DS as a continuous variable. The *Cronbach's* α coefficient for the CES-D-10 is 0.761 in 2013, 0.798 in 2015, and 0.804 in 2018.

#### Measurement of social engagement

Questions including “Have you done any of these activities in the last month” and “Frequency of activity in the last month” were used to measure the SE of participants. The former question includes 12 options: (1) Interacted with friends; (2) Played Ma-jong, chess, or cards, or went to a community club; (3) Provided help to family, friends, or neighbors who did not live with you and who did not pay you for the help; (4) Went to a sport, social, or other kinds of club; (5) Took part in a community-related organization; (6) Did voluntary or charity work; (7) Cared for a sick or disabled adult who does not live with you and who did not pay you for the help; (8) Attended an educational or training course; (9) Invested in stocks; (10) Used the Internet; (11) other; (12) None of these. Participants who chose options 1-11 of the former question were qualified to answer the latter question which includes three options: (1) Almost daily; (2) Almost every week; (3) Not regularly.

In our study, we combined the information mentioned above to form a continuous variable. Participants who chose options “(1) Almost daily, (2) Almost every week, (3) Not regularly” of the latter question were recorded as 3, 2, and 1 point, respectively. And those choosing option 12 were recorded as 0 points. The *Cronbach's* α coefficient of SE is 0.591 in 2011, 0.601 in 2013, and 0.609 in 2015. We also performed exploratory factor analysis, and detailed results of it were provided in Supplementary material 1. Based on the results of *Cronbach's* α coefficient and exploratory factor analysis, we may conclude that the measurements of SE used in this study were reliable.

#### Identification of covariate

Following previously published studies (Lei et al., 2014), we considered potentially covariates associated with DS disparity. Possible covariates include gender; residential region; education level; marital status; wearing dentures; chronic disease; satisfaction; insurance; sleeping time; nap time; eyesight; drinking; hearing; smoking. Details about the covariates assignment table are in Supplemental material 2.

Chronic diseases included hypertension, dyslipidemia, diabetes or high blood sugar, cancer or malignant tumor, chronic lung disease, liver disease, heart problems, stroke, kidney disease, stomach or other digestive diseases, emotional or nervous or psychiatric problems, memory-related disease, arthritis or rheumatism, and asthma. Chronic disease, eyesight, and hearing information were obtained by self-reporting.

Self-reported life satisfaction was also taken into consideration. The CHARLS used the problem “Please think about your life-as-a-whole. How satisfied are you with it” to measure satisfaction. In our study, this item was replaced by the variable “satisfaction” in short.

Although covariates including “income,” “the type of jobs,” “retirement,” “pain,” “physical activity,” and “cognition” were available in the database, however, they were dropped out because the missing rates are too high. Missing rates of these covariates are 63.97, 89.38, 20.69, 71.51, 65.91, and 37.36% in order.

### Statistical analysis

#### Missing data imputation

According to previous literature (Li et al., 2021b), more than 20% of the missing follow-up data, even if filled, the bias is relatively large. So, covariates with more than 20% missing values, including “income,” “work,” “retirement,” “pain,” “physical activity,” and “cognition,” were dropped out, and covariates with <20% missing values were imputed.

Since the K nearest-neighbors imputation (KNN) method is almost unaffected by the distribution of data and is suitable for categorical variables (Beretta and Santaniello, 2016), in this study it was used to impute missing data. The algorithm was implemented with R package “DMwR2” version 0.0.2 (Torgo, 2016). Based on previous research, when using KNN to fill in longitudinal data, imputation parameter *k* should be >10, so the parameter of KNN imputation was set as “*k* = 11,” which means the nearest 11 records were used (Beretta and Santaniello, 2016).

#### Description and comparison of basic characteristics

Count (proportion) was used for the presentation of categorical variables and mean (SD) for continuous variables. For comparison of characteristics across different years, analysis of variance was used for continuous variables, chi-square tests were applied for categorical variables, and nonparametric tests were used for ordinal variables.

#### Causal inference

Under the frame of causal inference, we can control confounding variables well and then more accurately estimate the association between SE and DS. In our analysis, confounders were identified by DAG (directed acyclic graphs). DAG is a tool for causal studies. And causal relationships are represented by arrows between the variables, pointing from cause to effect (Williams et al., 2018).

The specific process for determining the confounders is as follows: (1) using the linear mixed-effects regression (LMER) model to determine the associations among variables; (2) using DAG to visualize the relationships of variables; (3) using the Backdoor Criterion to identify confounders: (1) no vertex in confounders is a descendent of SE, and (2) confounders d-separates every path between SE and DS that has an incoming arrow into SE (backdoor path). Once confounding factors have been identified, the propensity score (PS) can be estimated.

In this study, the causal inference analysis (the control of confounders) was conducted through the propensity score (PS) method. PS method, which can be expressed as a function of multiple covariates as in equation 1, was first proposed by Rosenbaum and Rubin (Rosenbaum and Rubin, 1983). PS is the conditional probability of the *i*-th individual entering the observation group calculated based on the value of the known confounders (Rosenbaum and Rubin, 1983). In equation 2, the function *P(X)* is called the propensity score, that is, the propensity toward exposure to treatment 1 given the observed covariates *x*.


(1) $$\begin{array}{l} PS=P\left(X\right)=P\left(T=1|X\right) \end{array}$$

Under the frame of linear mixed-effects regression (LMER), we included the estimated PS as a covariate to perform causal inference (to control the confounders) (Vansteelandt and Daniel, 2014) and then estimated the association between DS and ES among all participants.

#### Subgroup identification analysis

The longitudinal data obtained from the CHARLS database includes a baseline (2011) and three follow-up waves (2013, 2015, and 2018) of the survey, thus subgroup identification must consider the longitudinal structure of the data. To solve this issue, we used the generalized linear mixed-effects model trees (GLMM trees) algorithm for subgroup identification analysis which allows for taking the longitudinal structure into account.

The GLMM tree algorithm builds on model-based recursive partitioning (MOB) which uses a parameter instability test for choosing partitioning variables (Fokkema et al., 2018). Because traditional MOB could not deal with longitudinal data, the GLMM tree was developed to take random effects into consideration (Fokkema et al., 2018).

The Generalized linear model (GLM) tree is a node-specific model. To estimate a GLMM tree, an iterative approach is taken (Fokkema et al., 2018). The interactive approaches alternate between (1) assuming random effects known, allowing for an estimate of fixed-effects by the GLM tree, and (2) assuming the GLM tree known, allowing for estimation of the random-effects parameter by GLMM (Fokkema et al., 2018).

The model can be expressed as in equation (2). In which, β_*j*_ is fixed effect whose value depends on terminal node *j, g* () is the link function, in this study the link function is defined as identity. The subscript *i* denotes individual observation, and *x*_*i*_ represents the column vector of fixed-effects predictor variables of observation *i* (variable details were presented in Supplemental material 1), and the random effects *b* are estimated globally.


(2) $$\begin{array}{l} g\left(\mu_{ij}\right)=x_{i}^{T}\beta_{j}+z_{i}^{T}b \end{array}$$

The standardized average probability difference (*Cohen's d*) of DS in the terminal node (*j*) is estimated by the method of the GLMM tree. The practical meaning of *Cohen's d* is that, for people belonging to a specific subgroup in the terminal node (*j*), there is a difference (which can be measured by *Cohen's d*) in the DS scores between those who have different scores of SE.

We incorporated the potential partition variables into the GLMM tree model and then get the *Cohen's d* in all subgroups explored by the GLMM tree. Since the GLMM tree can only explore subgroups with stronger associations between SE and DS while it cannot control confounders effectively, it is necessary to estimate the association between SE and DS under the frame of causal inference.

So, once the subgroups were identified by the GLMM tree, we would stratify the whole sample into different subgroups and test the association between DS and ES among subgroups identified by the GLMM tree. Finally, compare the differences in results between subgroups and the whole population.

Because women are generally at greater risk of DS (Gater et al., 1998), but are more likely to be engaged in social activities than men (Bernard, 1982), we clarified the gender differences in the associations between SE and DS.

In this study, the function “lmertree()” nested R package “glmertree” version 0.2-0 (Fokkema et al., 2018) was used to estimate the GLMM tree model and conduct the subgroup identification analysis. The waves of subjects were specified using the cluster argument of the “lmertree()” function. In our analysis, the level of significance was set as 0.05.

#### Statistical software

All statistical analysis was performed using the R programming language (R Core Team, 2021) and RStudio (Version 1.1.463, RStudio Inc., 250 Northern Ave, Boston, MA 02210) software.

## Results

### Basic characteristics

In total, 24,330 responders (13,521 senior adults were non-duplicate individuals) were included in our analysis and the median follow-up time is 4 years. Table 1 presents the basic characteristics of the responders in different years (survey waves). The results show that the majority of the participants lived in rural areas and were married. The mean SE scores of observations were 1.44 (*SD* 1.910) in 2011, 1.87 (*SD* 2.306) in 2013, and 1.92 (*SD* 2.505) in 2015, respectively, (Table 1). The mean DS scores of observations were 7.98 (*SD* 5.794) in 2013, 8.16 (*SD* 6.431) in 2015, and 8.88 (*SD* 2.505) in 2018. Detailed information are presented in Table 1.

**Table 1:** The basic characteristic of the study population.

**Characteristics**		**Year**			***P-value***
		**2011**	**2013**	**2015**	
		***n* (%)**	***n* (%)**	***n* (%)**	
**NO**.		5,716 (100)	8,491 (100)	10,123 (100)	
Residential region^a^ (N, %)	Rural	4,704 (82.3)	6,742 (79.4)	8,396 (82.9)	<0.001
	Urban	1,012 (17.7)	1,749 (20.6)	1,727 (17.1)	
Gender^a^ (N, %)	Male	2,716 (47.5)	4,068 (47.9)	4,875 (48.2)	0.442
	Female	3,000 (52.5)	4,423 (52.1)	5,248 (51.8)	
Education level^c^ (N, %)	Primary school graduate or below	4,063 (71.1)	5,853 (68.9)	6,893 (68.1)	<0.001
	Middle school, high school, or technical secondary school	1,580 (27.6)	2,473 (29.1)	3,045 (30.1)	
	Undergraduate or above	73 (1.3)	165 (1.9)	185 (1.8)	
Marital status^b^ (N, %)	Married	4,983 (87.2)	7,392 (87.1)	8,843 (87.4)	0.060
	Separated	34 (0.6)	30 (0.4)	29 (0.3)	
	Divorced	699 (12.2)	1,069 (12.6)	1,251 (12.4)	
Wearing denture^a^ (N, %)	Yes	509 (8.9)	435 (5.1)	431 (4.3)	<0.001
	No	5,207 (91.1)	8,056 (94.9)	9,692 (95.7)	
Chronic disease^c^ (N, %)	No	3,928 (68.7)	5,603 (66.0)	7,154 (70.7)	<0.001
	One	1,022 (17.9)	1,574 (18.5)	1,628 (16.1)	
	Two or more	766 (13.4)	1,314 (15.5)	1,341 (13.2)	
Insurance^a^ (N, %)	Yes	5,327 (93.2)	8,179 (96.3)	9,833 (97.1)	<0.001
	No	389 (6.8)	312 (3.7)	290 (2.9)	
Sleeping time^a^ (N, %)	<6 h	1,672 (29.3)	2,884 (34.0)	3,122 (30.8)	<0.001
	6 h or more	4,044 (70.7)	5,607 (66.0)	7,001 (69.2)	
Naptime^a^ (N, %)	<30 min	3,348 (58.6)	4,435 (52.2)	5,065 (50.0)	<0.001
	30 min or more	2,368 (41.4)	4,056 (47.8)	5,058 (50.0)	
Eyesight^c^ (N, %)	Good	716 (12.5)	1,431 (16.9)	1,637 (16.2)	<0.001
	Fair	1,365 (23.9)	1,640 (19.3)	1,517 (15.0)	
	Poor	3,635 (63.6)	5,420 (63.8)	6,969 (68.8)	
Drinking^a^ (N, %)	Yes	1,896 (33.2)	2,905 (34.2)	3,579 (35.4)	0.018
	No	3,820 (66.8)	5,586 (65.8)	6,544 (64.6)	
Hearing^c^ (N, %)	Good	829 (14.5)	1,543 (18.2)	1,870 (18.5)	<0.001
	Fair	1,672 (29.3)	1,926 (22.7)	1,846 (18.3)	
	Poor	3,215 (56.2)	5,022 (59.1)	6,405 (63.3)	
Smoking^b^ (N, %)	Never smoke	3,450 (60.4)	6,460 (76.1)	5,979 (59.1)	<0.001
	Still smoke	1,775 (31.1)	1,457 (17.2)	2,858 (28.2)	
	Totally quit	491 (8.6)	574 (6.8)	1,286 (12.7)	
Age^c^ (N, %)	45–59	3,127 (54.7)	2,786 (32.8)	5,088 (50.3)	<0.001
	60–79	2,426 (42.4)	4,879 (57.5)	4,693 (46.4)	
	Over 80	163 (2.9)	826 (9.7)	342 (3.4)	
SE^d^ (Mean ± SD)		1.44 ± 1.910	1.87 ± 2.306	1.92 ± 2.505	<0.001
		**Year**			
DS^d^ (Mean ± SD)		2013	2015	2018	<0.001
		7.98 ± 5.794	8.16 ± 6.431	8.88 ± 6.928	

a Chi-square test using a 3 × 2 table.

b Chi-square test using a 3 × 3 table.

c Nonparametric tests.

d Analysis of variance. SE, social engagement; DS, depressive symptoms.

### Identification of confounders

**Figure 2:**
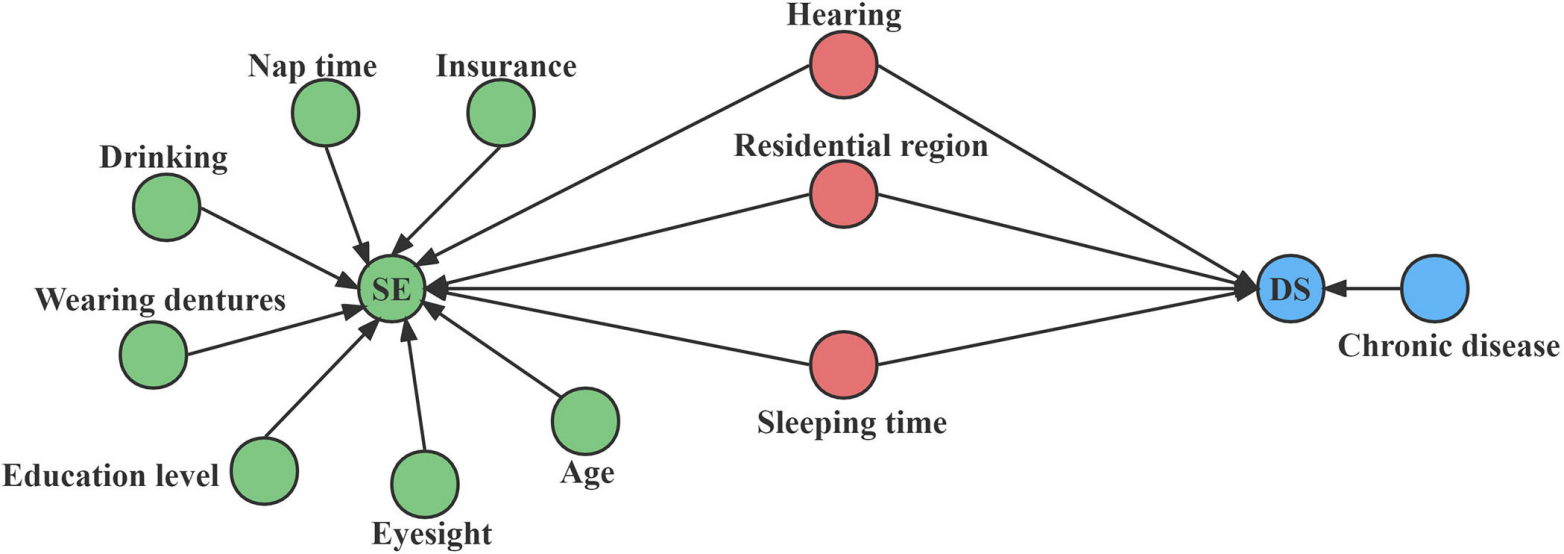
DAG analysis for selecting confounding variables. Causal relationships are represented by arrows between the variables, pointing from cause to effect. The green circle represents SE and its associated covariates, the blue circle represents DS and its related covariates, and the red circle represents the confounding variables.

DAG was used to represent and better understand the associations between factors and outcomes (Figure 2). According to the DAG and the backdoor criterion, variables including residential region, hearing, and sleeping time were identified as confounders.

### Overall analysis under causal inference frame

The propensity score was then estimated with the identified confounders. With the GLMM analysis, we found the association between DS and SE was statistically significant (*P* = 0.016) among all individuals under the causal inference frame and the odds ratio (OR) was 0.957 (95%*CI*: 0.923–0.992). Detailed results are presented in Table 2.

**Table 2:** Subgroups analysis and estimation of the OR between SE and DS with LMER under causal inference frame.

**Population**	***Cohen's d* ^a^**	**OR (SE)**	**95%CI (SE)**	***P*-value (SE)**
Overall analysis with whole samples	**/**	0.957	(0.923, 0.992)	0.016
Subgroup a	−0.1288313	0.897	(0.830, 0.971)	0.007
Subgroup b	0.0008699542	1.008	(0.960, 1.059)	0.742
Subgroup c	−0.09828412	0.916	(0.854, 0.981)	0.013
Male	/	0.935	(0.888, 0.985)	0.011
Female	/	0.979	(0.931, 1.029)	0.399

OR, odds ratio; CI, confidence interval; SE, social engagement. The symbol alphabet

a means the standardized average probability difference of DS in the terminal nodes.

### Subgroup identification

**Figure 3:**
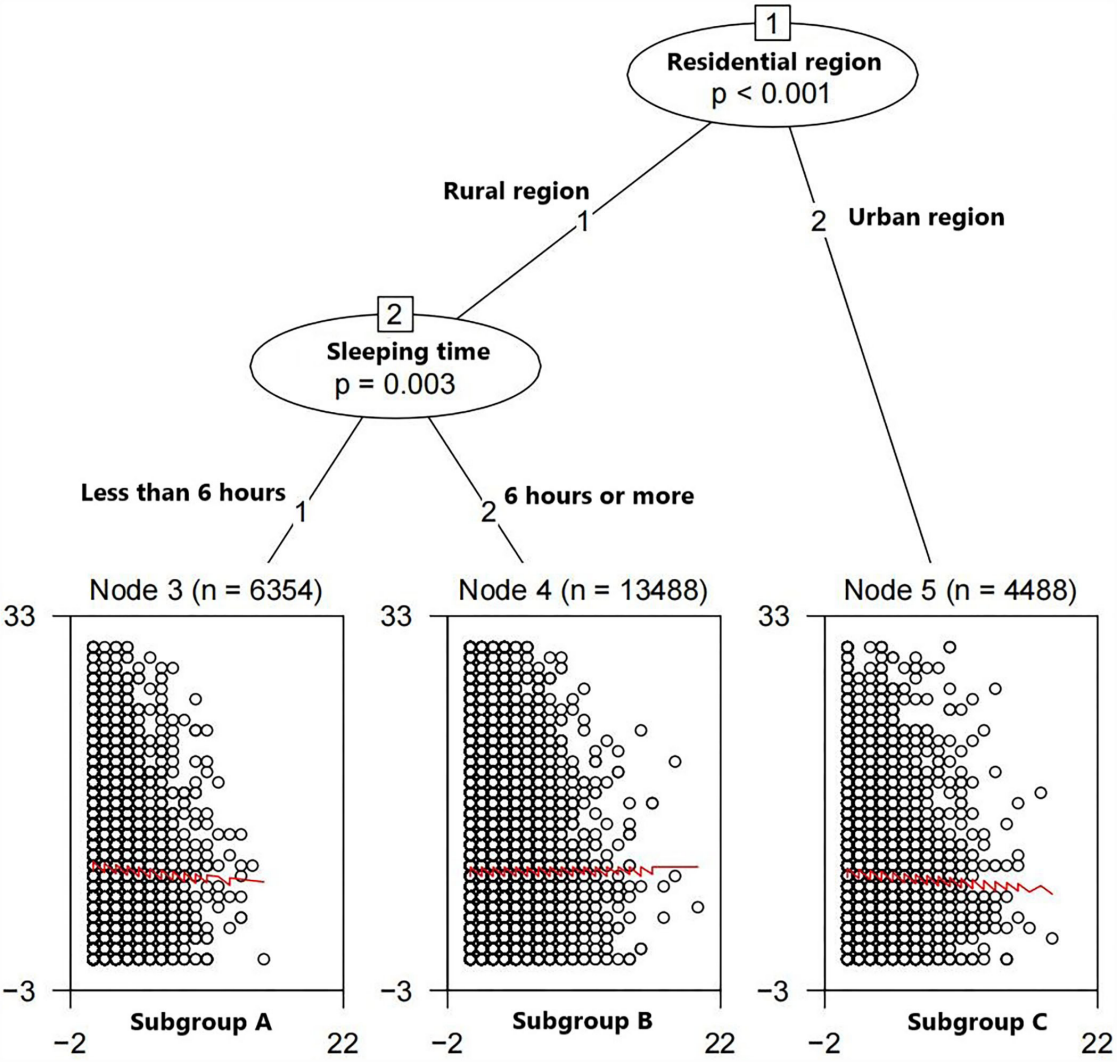
GLMM tree fitted. The selected partitioning variables are residential region and level of sleeping time. Terminal nodes: y axes represent CES.D-10 scores, and x axes represent SE scores.

The subgroup identification analysis was conducted through the GLMM tree, and the survey wave was defined as the random effect. We incorporated all possible partition variables, including “Hearing,” “Residential region,” “Sleeping time,” “Gender,” “Insurance,” “Naptime,” “Drinking,” “Wearing dentures,” “Education level,” “Marital status,” “Eyesight,” “Smoking,” “Chronic disease,” and “Age,” into the tree building process. The GLMM tree (Figure 3) selected “Residential region” as the first partitioning variable and “Sleeping time” as the second partitioning variable.

The terminal nodes presented in Figure 3 show a double factor–subgroup interaction: for participants who live in rural areas and sleep <6 h per day (Subgroup A), improving SE scores can reduce the DS scores (*Cohen's d* = −0.1288313). For people who live in rural areas and sleep more than 6 h per day (Subgroup B), SE can provide more or less the same change in DS scores (*Cohen's d* = 0.0008699542). For individuals who live in urban areas (Subgroup C), high SE scores can lead to low DS scores (*Cohen's d* = −0.09828412).

### Subgroup analysis under causal inference frame

In subgroups A and C, the associations between DS and SE are also statistically significant under the causal inference frame. ORs of these two subgroups are 0.897 (95%*CI*: 0.830–0.971) and 0.916 (95%*CI*: 0.854–0.981), respectively, as shown in Table 2. The associations between SE and DS in these subgroups are more significant than in the whole samples; similarly, their ORs are smaller.

In subgroup B, the association between DS and SE is not statistically significant under the causal inference frame (*P* = 0.742), which is different from the whole people (Table 2).

The associations between DS and SE are statistically significant in males (OR: 0.935, *P* = 0.011, 95%CI: 0.888–0.985) but not statistically significant in females (OR: 0.979, *P* = 0.399, 95%CI: 0.931–1.029).

## Discussion

Preventing DS is an important prerequisite for improving the quality of life, especially for middle-aged and senior citizens. Published studies indicated that SE was closely related to DS and could act as a key factor in the prevention of DS (Solomonov et al., 2019; Bae, 2021). Results of previously published studies indicated that there were differences in the preventive effects of SE on depression in people with different characteristics (Min et al., 2016). Therefore, in order to deeper understand the heterogeneity of effects of the SE in subgroups of people with different characteristics, we launched this study. In general, we found that SE was associated with decreased risk of DS. Furthermore, in subgroup analysis, we found that SE may be more effective in preventing DS for people who live in the rural area with <6 h of sleep per day, and who live in the urban area. However, for people who live in rural areas and sleep more than 6 h per day, we did not find a significant association between SE and DS.

Sleep disturbances increase the risk of depression. The published study suggested that individuals with severe insomnia are more likely to develop depression (Schramm et al., 1995). In China, Jiang et al. (2020) used data from Henan Rural Cohort and found that short night sleep duration (<6 h) was the risk factor for DS in rural regions (Jiang et al., 2020). At the same time, sleep disorders are a typical symptom of most people with depression (Fang et al., 2019). In other words, depression can lead to sleep disturbances. In addition, manipulation of sleep–wake cycles, such as sleep deprivation or early sleep periods, can alleviate depressive symptoms. This evidence suggests a strong bidirectional relationship between sleep, sleep changes, and depression (Riemann et al., 2001).

In addition, sleep is correlated with both the quantity and quality of social relationships (Gordon et al., 2021), and lacking sleep leads to an increase in loneliness and social isolation (ben Simon and Walker, 2018). Ben Simon and Walker conducted a longitudinal study and demonstrated that insufficient sleep leads to a neural and behavioral phenotype of social withdrawal and loneliness (ben Simon and Walker, 2018). Moreover, daytime sleepiness caused by poor sleep quality at night also increases social withdrawal (Holding et al., 2020). Therefore, a vicious cycle of sleep deprivation, daytime sleepiness, and social withdrawal may be key causes of depression. Increasing social interaction or improving sleep quality may be a key measure to break the vicious cycle and, by extension, prevent DS. However, rural residents with sufficient sleep time (>6 h) were not in this vicious circle, and our study did not confirm the association between SE and DS in this subgroup. Therefore, further research may be needed to further explore the association between SE and DS in this particular subgroup.

Retirement problems may cause the results that SE is more effective among the urban elderly in preventing DS. Unlike the rural residents engaged in agricultural work which was characterized by a “small-scale peasant economy” (based on the family as a unit and individual ownership of the means of production, which relies entirely or mainly on its own labor to meet its own consumption), the aged people in urban are more likely to face retirement problems. Sudden changes in social networks (caused by retiring) may make them out of their comfort zone and increase the risk of DS. The result of a systematic review and meta-analysis containing a total of 25 longitudinal studies between 1980 and 2020 showed that retirement is associated with more DS (Li et al., 2021a). Essentially, a possible cause for this is the change in the social relationship after retiring. More specifically, retirement is a process of losing or weakening a working (or social) role (Riley et al., 1994), and people are forced to change their life roles, which can cause psychological distress (Rohwedder and Willis, 2010). Therefore, increased SE can improve their mental health, and previous studies have shown that volunteering or working after retirement can be beneficial to mental health (Chen, 2012).

As for middle-aged adults in China, a previous study has found that SE can reduce the incidence of DS (Bhattacharya et al., 2016). Middle age is a special period in life when they shift their focus from exploring new social relationships to cultivating existing ones (Kiesow et al., 2021), such as interacting with close family members or friends (Bhattacharya et al., 2016). Therefore, maybe a good social connection with families or friends can help middle-aged people to enhance good mental health and prevent DS.

Previous studies have shown that DS is more common in women than men (Altemus et al., 2014), and our study confirms this conclusion. Besides, we also found that SE reduced the risk of depression in men, but not in women. While women are more likely to participate in social activities than men, a study that separates social networks' stress and support components found that the former is more closely related to women's mental health than the latter (Bernard, 1982). This suggests that attending social events makes it easier for women to get stress rather than stress relief. Therefore, men should be more involved in social activities than women to reduce the incidence of DS. In addition, it is recommended that families provide positive feedback in daily life to reduce the pressure that social activity places on women.

In our study, we found that from 2013 to 2018, the scores of DS showed an upward trend. With the rapid development of China's society and economy and the increasing degree of aging, the incidence of depression among the middle-aged and elderly Chinese is inevitable and may continue to rise in the future (United Nations, 2019). It is urgent to solve the problem of depression in the middle-aged and elderly Chinese. However, China has a large aging population, so the implementation of full coverage of the prevention is bound to bring a huge public health burden. Therefore, cost-effective interventions are important for the prevention of DS.

Our findings suggest that enhancing SE may be useful for reducing the risks of DS for the middle-aged and elderly in China, and generally speaking, SE is a part of everyday life and a modifiable factor in daily life. The cost of enhancing the SE of people through family and community efforts is relatively low. Thus, we could conclude that enhancing SE may be potentially a cost-effective way in preventing the initiation or the progression of DS.

Besides, in this study, we also found that two subgroups of people, including those rural people who sleep <6 h per day and those who live in urban areas, may benefit more in preventing DS or relieving the severity of it through improving SE. From the perspective of precision prevention, if sufficient attention can be given to these two subgroups, better DS prevention effects may be obtained and resource savings can be achieved. Therefore, in order to accurately prevent DS, communities and families are supposed to maintain a good community environment to ensure that there is a good environment suitable for the middle-aged and elderly adults to sleep at night. Besides, families are suggested to provide maximum social support to the middle-aged and elderly members, and the middle-aged and elderly people should also actively take measures to deal with the problem of retirement, develop good living styles, and avoid sleeping loss.

At present, the definition of SE varies, and a review article defines SE as “the action of being involved in community life, socially or politically and structured by the environment, which places can be shared, and which are significant” by summarizing the relevant literature from 2009 to 2020 (Levasseur et al., 2022). The inclusion of social activities in our study directly follows the relevant questions defined in the CHARLS questionnaire. It divided SE into four categories, including activities for the purpose of leisure, activities that aim to provide pro bono service, activities through the Internet, and other activities. The inclusion of social engagement in this study is consistent with the definition mentioned above.

This study has several strengths. First, the effects of SE and DS are not short term, and the longitudinal data structure could ensure the evaluation of the longtime effects. Therefore, the results of longitudinal studies are more reliable than cross-sectional studies. Then, this study used baseline and 3 years of follow-up data, including the follow-up data of 2018 newly published in 2020. With a large sample size, nationwide sampling, and a seven-year time span, the research objects of this study are more representative. Besides, in subgroup analysis, a large sample size prevented the problem of excessively sparse subgroups and ensure the robustness of the results. Furthermore, compared with linear models, tree-based methods that generate hypotheses are more suitable for exploratory research on subgroup identification, and the GLMM tree shows higher accuracy and statistical power in subgroup identification (Beretta and Santaniello, 2016). Finally, causal inference analysis has rapidly evolved for generating scientific evidence (Hernán and Robins, 2020) since confounders were almost unavoidable in observational studies. In this study, we applied the PS adjustment to control possible confounding factors, and the conclusions drawn under the framework of causal inference are reliable.

There are some limitations to this study. This study was conducted based on the observational survey data obtained from the CHARLS study, and a prospective interventional study is still needed to verify the association between SE and DS. Moreover, DS is not based on clinical diagnosis but on scale measurement. Although the CES-D-10 scale has been validated for its effectiveness in Chinese populations, the judgment of DS may be biased to some extent. Measurements of SE consist of a series of questions. Based on the results of *Cronbach's* α coefficient and exploratory factor analysis, we may conclude that the measurements of SE used in this study were reliable. In addition, variables “the type of jobs” and “physical activity” are related to the occurrence of DS, but the missing rates of them are 89.38% and 65.91%. Variables missing more than 20% are not recommended to be imputed (Li et al., 2021b); thus, these two covariates were excluded. Finally, the effects of different kinds of SE were not included in the study. In the future, it should be determined what kind of social activities can minimize DS and obtain more quantitative results on this issue.

## Conclusions

In general, we explored the association between SE and DS based on the longitudinal follow-up data obtained from the CHARLS study, and the results indicate that they are longitudinal and negatively associated. Our findings suggest that the enhanced SE may be useful for reducing the risks of DS for the middle-aged and elderly in China, especially for those middle-aged and elderly residents living in rural areas with less than six hours of sleep and those living in urban areas.

## Data availability statement

Publicly available datasets were analyzed in this study. This data can be found here: https://charls.charlsdata.com/pages/data/111/zh-cn.html.

## Author contributions

FC: the conception and design of this study, critical revision of the manuscript, and supervision of the study. YY and YL: data acquisition and management, statistical analysis, and interpretation of the results. YY: drafted the manuscript and assisted in the revision of the manuscript. PZ and JW: helped with data analysis. BM, LP, and YZ: assisted with the interpretation of the results and helped with the revision of the manuscript. All authors have reviewed and approved the manuscript before submission.

## Funding

This study has been supported by the National Natural Science Foundation of China (81703325).

## Conflict of interest

The authors declare that the research was conducted in the absence of any commercial or financial relationships that could be construed as a potential conflict of interest.

## Publisher's note

All claims expressed in this article are solely those of the authors and do not necessarily represent those of their affiliated organizations, or those of the publisher, the editors and the reviewers. Any product that may be evaluated in this article, or claim that may be made by its manufacturer, is not guaranteed or endorsed by the publisher.
