# Supplementary material for: The association between social engagement and depressive symptoms in middle-aged and elderly Chinese: A longitudinal subgroup identification analysis under causal inference frame
Source: Front Aging Neurosci. 2022 Sep 1;14:934801. doi: 10.3389/fnagi.2022.934801 (PMC9476863; doi:10.3389/fnagi.2022.934801)
Supplement: Supplementary file 1 [file Data_Sheet_1.docx]

| **Supplemental Material 1: Rotated component matrix of social engagement** | | | | | |
| --- | --- | --- | --- | --- | --- |
| **Year** | **Items** | **Factor 1** | **Factor 2** | **Factor 3** | **Factor 4** |
| **2011** | Interacted with friends | **0.642** | 0.291 | 0.162 | 0.246 |
|  | Played Ma-jong/cards/chess | **0.684** | 0.166 | 0.029 | 0.270 |
|  | Went to club | **0.429** | 0.171 | 0.038 | 0.218 |
|  | Took part in a community-related organization | **0.462** | 0.097 | 0.056 | 0.095 |
|  | Attended an educational or training course | **0.429** | 0.032 | 0.207 | 0.287 |
|  | Provided help to people who lived apart | **0.423** | **0.433** | **0.328** | 0.116 |
|  | Voluntary or charity | **0.485** | 0.274 | **0.380** | 0.245 |
|  | Cared for a sick or disabled adult who lived apart | **0.357** | **0.470** | **0.322** | 0.297 |
|  | Stock investment | 0.257 | 0.015 | 0.293 | **0.566** |
|  | Used the internet | 0.276 | 0.005 | 0.131 | **0.521** |
|  | Other | 0.007 | 0.008 | 0.067 | 0.026 |
| **2013** | Interacted with friends | **0.389** | 0.548 | 0.265 | 0.056 |
|  | Played Ma-jong/cards/chess | **0.346** | 0.431 | 0.282 | 0.267 |
|  | Went to club | **0.465** | 0.022 | 0.148 | 0.292 |
|  | Took part in a community-related organization | **0.507** | 0.129 | 0.100 | 0.131 |
|  | Attended an educational or training course | **0.389** | 0.275 | 0.292 | 0.236 |
|  | Provided help to people who lived apart | **0.474** | 0.385 | **0.354** | **0.405** |
|  | Voluntary or charity | **0.459** | 0.038 | **0.433** | **0.331** |
|  | Cared for a sick or disabled adult who lived apart | **0.306** | 0.206 | **0.368** | **0.590** |
|  | Stock investment | **0.365** | **0.467** | **0.456** | 0.217 |
|  | Used the internet | **0.551** | **0.414** | **0.345** | 0.155 |
|  | Other | 0.046 | 0.182 | 0.096 | 0.216 |
| **2015** | Interacted with friends | **0.409** | 0.222 | **0.483** | 0.012 |
|  | Played Ma-jong/cards/chess | **0.298** | 0.126 | **0.553** | **0.406** |
|  | Went to club | **0.450** | 0.067 | **0.362** | **0.315** |
|  | Took part in a community-related organization | **0.478** | 0.118 | **0.331** | **0.432** |
|  | Attended an educational or training course | **0.401** | 0.057 | **0.381** | 0.112 |
|  | Provided help to people who lived apart | **0.493** | **0.366** | 0.076 | **0.391** |
|  | Voluntary or charity | **0.491** | **0.335** | 0.155 | 0.204 |
|  | Cared for a sick or disabled adult who lived apart | **0.327** | **0.366** | 0.064 | **0.544** |
|  | Stock investment | **0.398** | **0.666** | 0.170 | 0.209 |
|  | Used the internet | **0.575** | **0.536** | 0.070 | 0.154 |
|  | Other | 0.169 | 0.085 | 0.398 | 0.131 |

| **Supplemental Material 2: The covariates assignment table.** | | |
| --- | --- | --- |
| **Covariates** | **Characteristics** | **Value** |
| **Residential Gender region** | rural | 1 |
|  | urban | 2 |
| **Gender** | male | 1 |
|  | female | 2 |
| **Education level** | primary school graduate or below | 1 |
|  | middle school, high school, or technical secondary school | 2 |
|  | undergraduate or above | 3 |
| **Marital status** | married | 1 |
|  | separated | 2 |
|  | divorced | 3 |
| **Wearing dentures** | yes | 1 |
|  | no | 2 |
| **Chronic disease** | no | 0 |
|  | one | 1 |
|  | two or more | 2 |
| **Insurance** | yes | 1 |
|  | no | 2 |
| **Sleeping time** | less than 6h | 1 |
|  | 6h or more | 2 |
| **Nap time** | less than 30min | 1 |
|  | 30min or more | 2 |
| **Eyesight** | good | 1 |
|  | fair | 2 |
|  | poor | 3 |
| **Drinking** | yes | 1 |
|  | no | 2 |
| **Hearing** | good | 1 |
|  | fair | 2 |
|  | poor | 3 |
| **Smoking** | never smoke | 1 |
|  | still smoke | 2 |
|  | totally quit | 3 |
| **Age** | 45-59 | 1 |
|  | 60-79 | 2 |
|  | over 80 | 3 |
